# Embolic Protection Devices in Transcatheter Aortic Valve Implantation: A Narrative Review of Current Evidence

**DOI:** 10.3390/jcm14124098

**Published:** 2025-06-10

**Authors:** George Latsios, Nikolaos Ktenopoulos, Anastasios Apostolos, Leonidas Koliastasis, Ioannis Kachrimanidis, Panayotis K. Vlachakis, Odysseas Katsaros, Emmanouil Mantzouranis, Sotirios Tsalamandris, Maria Drakopoulou, Andreas Synetos, Constantina Aggeli, Konstantinos Tsioufis, Konstantinos Toutouzas

**Affiliations:** First Department of Cardiology, National and Kapodistrian University of Athens, Hippokration General Hospital of Athens, 11527 Athens, Greece; glatsios@gmail.com (G.L.); anastasisapostolos@gmail.com (A.A.); lkoliastasis@gmail.com (L.K.); iskachrimanidis@gmail.com (I.K.); vlachakispanag@gmail.com (P.K.V.); odykatsaros@gmail.com (O.K.); mantzoup@gmail.com (E.M.); stsalamandris@hotmail.com (S.T.); mdrakopoulou@hotmail.com (M.D.); synetos@yahoo.com (A.S.); dina.aggeli@gmail.com (C.A.); ktsioufis@gmail.com (K.T.); ktoutouz@gmail.com (K.T.)

**Keywords:** TAVI, transcatheter aortic valve implantation, cerebral embolic protection, stroke, embolic protection devices, silent infarcts

## Abstract

Transcatheter aortic valve implantation (TAVI) has emerged as a transformative therapy for patients with severe aortic stenosis (AS) across all surgical risk groups. However, periprocedural cerebrovascular events (CVEs), including overt stroke and silent cerebral embolism, remain significant complications. As a result, the use of embolic protection devices (EPDs) during TAVI has been proposed to mitigate this risk. Our aim was to provide a comprehensive review of the current evidence on the efficacy, safety, and clinical utility of embolic protection devices in TAVI procedures. According to the existing literature, EPDs are effective in capturing embolic debris during TAVI and are associated with a reduction in silent cerebral lesions as detected by diffusion-weighted MRI. While some RCTs and meta-analyses demonstrate a potential benefit in reducing disabling stroke, evidence for a consistent reduction in overall stroke or mortality remains inconclusive. Subgroup analyses suggest the greatest benefit in patients at elevated stroke risk, while current-generation EPDs demonstrate high technical success and an acceptable safety profile. Subsequently, EPDs represent a promising adjunct to TAVI, particularly in high-risk populations. However, routine use in all patients is not yet supported by consistent clinical evidence. Further large-scale trials and long-term outcome data are needed to clarify their role in improving neurological outcomes and to guide selective patient application.

## 1. Introduction

Transcatheter aortic valve implantation (TAVI) has become an established therapeutic modality for patients with symptomatic aortic stenosis (AS) across the spectrum of surgical risk [1,2,3]. Degenerative aortic stenosis (AS) is not only a chronic, slowly progressive valve disease manifested by the classic triad of exertional angina, syncope and heart-failure dyspnea, but, in up to one-fifth of cases, the first clinical contact is an acute decompensation: flash pulmonary oedema, low-output cardiogenic shock or refractory New York Heart Association class IV dyspnea that mandates urgent hospitalization. Pathophysiologically, even minor shifts in preload/afterload can overwhelm a hypertrophied, non-compliant left ventricle, leading to precipitous hemodynamic collapse; prognostically, such presentations carry a dismal early-mortality risk and remain strong predictors of adverse events even after definitive transcatheter or surgical valve replacement. Recognizing this acute phenotype of AS underscores why contemporary TAVI programs must integrate both hemodynamic stabilization and cerebral-embolism-prevention strategies [4].

As the application of TAVI expands to an increasing number of individuals, minimizing potential complications is of paramount importance [5,6,7]. Among these complications, cerebrovascular events (CVEs), particularly stroke, remain a significant concern, associated with considerable morbidity and mortality [8]. While the reported incidence of stroke following TAVI varies from 1% to 11%, with a median of approximately 4%, contemporary stroke rates are observed in the range of 2% to 4% [9,10]. Despite advancements in both TAVI implantation techniques and the design of the devices utilized [11], a substantial reduction in stroke rates has not been consistently observed, suggesting the need for adjunctive strategies to mitigate this risk [12]. Furthermore, the expanding application of TAVI to lower-risk, often younger, patients with longer life expectancies underscores the importance of preventing even subtle neurological damage that could manifest over time [2,13,14,15].

Studies utilizing magnetic resonance imaging (MRI) or cerebral filters have demonstrated that brain embolization occurs in the vast majority of patients undergoing TAVI, even in the absence of clinically apparent neurological deficits [8,16]. These so-called silent cerebral infarctions have been reported in a wide range, from 58% to 100% of patients [17]. Embolization during the interventions in the valve is recognized as a primary mechanism contributing to strokes in this patient population [17]. These silent lesions are not considered benign, as they have been linked to an increased risk of postprocedural delirium, dementia, and long-term neurocognitive decline [17,18]. To address this critical issue, embolic protection devices (EPDs) have been developed. These devices function as a mechanical barrier, aiming to prevent embolic debris from reaching the cerebral vasculature [19]. Their primary goal is to capture or deflect these emboli generated during the TAVI procedure, thereby reducing the risk of peri-procedural ischemic events [19]. The high frequency of silent embolization, even when overt stroke does not occur, suggests potential for subtle, yet significant, neurological consequences that EPDs are designed to address [20].

We searched PubMed/MEDLINE, Embase and Cochrane CENTRAL databases for this narrative review using the terms “TAVI”, “TAVR”, “stroke” and “cerebral embolic protection”. No language restrictions were applied. The objective of this review is to provide a comprehensive overview of the current knowledge regarding the use of EPDs in TAVI. This includes an examination of the available evidence concerning their impact on histopathological findings, cerebral imaging outcomes, and neurological events, as well as a synthesis of the clinicopathological rationale and the evidence supporting their use. While a new meta-analysis is beyond the scope of this review, we synthesized the findings from existing meta-analyses to provide an updated understanding of the effect of cerebral EPDs during TAVI on various clinical, neurological, and safety parameters [21].

## 2. Understanding the Causes and Timing of Stroke in TAVI Procedures: The Role of TAVI-Induced Debris

**Figure 1:**
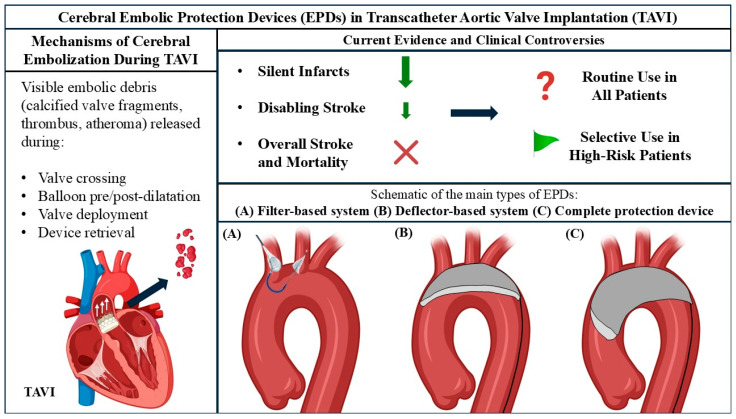
Cerebral embolic protection in TAVI. This illustration summarizes the pathophysiology of cerebral embolization during transcatheter aortic valve implantation (TAVI), the types and mechanisms of cerebral embolic protection devices (EPDs), and the current clinical evidence regarding their use. It depicts the release of embolic debris during key procedural steps of TAVI and its potential to cause overt stroke or silent cerebral infarcts. Alongside, it illustrates the three primary categories of EPDs [(**A**) Filter-based system; (**B**) Deflector-based system; (**C**) Complete protection device] and the current evidence and ongoing controversies regarding the clinical efficacy of EPDs. While EPDs effectively reduce the volume of silent brain infarcts and may lower the incidence of disabling stroke in selected patients, no consistent reduction in overall stroke rates or mortality has been demonstrated. The routine use of EPDs remains debated, with current guidelines recommending a selective approach pending results from ongoing large-scale randomized trials.

The process of TAVI can lead to the release of various types of debris into the circulation [9]. This embolic material can originate from several sources, including fragments of the arterial wall, thrombi, valve tissue, and even foreign bodies introduced during the procedure [9] (Figure 1). Specific components of this debris have been identified as cholesterol particles, air, atherosclerotic plaque material, thrombus, and calcified valve material [22,23,24]. The disruption of existing atheromatous or calcific deposits during different stages of the TAVI procedure is the primary mechanism that provokes these athero- or thrombo-embolic events [9]. Notably, certain procedural aspects, such as the repositioning of the transcatheter heart valve (THV), have been associated with a greater quantity of captured debris [9,25]. Furthermore, patients with bicuspid aortic valves appear to have a higher likelihood of experiencing embolization of larger particles [26]. Histopathological examination of the material captured by filter-based EPDs has revealed a heterogeneous composition, including acute thrombus, remnants of the arterial wall, valve tissue, calcific fragments, myocardial tissue, and occasionally, foreign material [9,27]. The diverse nature of this debris suggests multiple points of origin during the TAVI procedure, and the increased risk associated with specific anatomical features or procedural maneuvers underscores the complexity of achieving effective embolic protection [20].

The majority of periprocedural strokes following TAVI occur within a relatively short timeframe after the intervention [19]. Approximately 50% of these events are observed within the first 72 h, and about 80% manifest within the first week following the procedure [28]. Embolic events can occur during various critical steps of the TAVI procedure, including the navigation of the delivery system through the aorta towards the aortic annulus, the precise positioning and subsequent displacement of the calcified native aortic valve by the new THV, and any further manipulations, such as post-dilatation, aimed at optimizing the final result [29]. Furthermore, clinical symptoms of stroke often become apparent once the effects of periprocedural anticoagulation begin to wane [30]. The early temporal clustering of most embolic strokes emphasizes the critical need for immediate cerebral protection during the periprocedural period, which is the primary rationale for the use of EPDs [19].

Stroke definitions used in this review follow the Valve Academic Research Consortium-3 (VARC-3) nomenclature. VARC-3 classifies any new, vascular-origin neurological deficit lasting ≥24 h (or causing death) as an overt stroke, which is further stratified into disabling (modified Rankin Scale > 2 at 90 days or NIHSS ≥ 6 at 24 h) and non-disabling events. Transient ischemic attack (TIA) and clinically silent diffusion-weighted-MRI lesions are captured as separate categories, recognizing their different prognostic weights. This harmonized hierarchy allows meaningful comparisons across TAVI trials and keeps the focus on strokes that translate into long-term functional disability—the outcome most likely to be influenced by cerebral embolic protection [31].

Interestingly, the type of prosthetic valve used during TAVI appears to influence the characteristics of the captured debris. For example, the mechanically expandable Lotus valve has been associated with less debris originating from the native aortic valve, but a greater amount of calcific, endothelial, and myocardial tissue compared to the balloon-expandable SAPIEN 3 and the self-expanding EVOLUT R/PRO valves [32,33]. These findings highlight that the specific valve type is a factor that can affect both the quantity, and the size distribution of the embolic particles released during the procedure [32]. The insights gained from histopathological analysis are invaluable, providing a deeper understanding of the composition and size range of the emboli generated during TAVI [9]. This information can be used to guide the development of more effective EPDs and to refine procedural techniques with the goal of minimizing embolic burden.

Alongside, there is the procedure-level embolic risk. Continuous transcranial Doppler monitoring has shown that intracranial high-intensity transient signals (HITS) spike during valve positioning and final deployment, accounting for ~60% of the total cerebral embolic load, whereas catheter passage across the aortic arch and initial balloon aortic valvuloplasty contribute 15% and 18%, respectively. In corroborative DW-MRI series, the number and volume of new lesions parallel this distribution. Clinically, contemporary multicenter registries still report a 30-day major stroke rate of 2–3% and minor stroke of 1–2% despite newer low-profile valves and streamlined delivery systems. These observations highlight that technique optimization—minimal manipulation, “direct-TAVI” without routine pre-dilation when feasible, strict ACT control, and selective use of cerebral embolic protection—remains pivotal to stroke prevention [34,35,36].

Furthermore, considering left bundle branch block (LBBB) and cerebrovascular events, the available data indicate that new-onset LBBB after TAVI—whether persistent or transient/temporary—does not translate into a higher risk of clinical stroke. In a multicenter cohort of 668 balloon-expandable TAVI recipients, the incidence of 30-day stroke was comparable in patients with persistent LBBB versus those with intact conduction (5.1% vs. 2.4%, *p* = 0.25), and no excess cerebrovascular events emerged during 13-month follow-up. Complementing these findings, a 2022 single-center study that specifically tracked temporary LBBB resolving before discharge likewise reported no difference in periprocedural or one-year stroke rates between groups (0.6% vs. 0.8%, *p* = 0.79). Taken together, the current evidence does not support using LBBB alone as a criterion for deploying cerebral embolic protection [37,38].

To summarize, the clinical consequences of embolic events during TAVI can range from devastating overt strokes to more subtle silent cerebral emboli. Overt stroke remains a major concern, associated with a significantly increased risk of mortality and long-term disability [39]. Beyond clinically evident stroke, the frequent occurrence of silent brain infarcts, detected through diffusion-weighted MRI (DW-MRI), also raises concerns [17], since they have been linked to an increased risk of postprocedural neurological adverse events [17,40]. Emerging evidence suggests that these silent brain lesions may not be entirely benign, and their potential impact over the long term, particularly in younger patients undergoing TAVI, warrants careful consideration [12]. While overt stroke represents the most immediate and severe complication, the high prevalence and potential long-term neurological consequences associated with silent cerebral emboli underscore the importance of striving for comprehensive cerebral protection during TAVI [24,41]. Addressing both overt and silent embolic events is crucial for optimizing the overall outcomes of TAVI procedures [42].

## 3. Cerebral Embolic Protection Device Types

Embolic protection devices employed during TAVI can be broadly categorized into two main types based on their mechanism of action: filter-based devices and deflector-based devices, producing partial or complete protection [43] (Figure 1). Filter devices are designed to capture embolic material as blood flows through them, effectively trapping the debris before it can reach the cerebral circulation. A key advantage of these filter systems is the ability to retrieve the captured embolic material, allowing for subsequent histopathological analysis to determine its composition and origin [27]. In contrast, deflector-type systems are positioned in the aortic arch with the aim of redirecting embolic debris away from the cerebral vessels. These devices typically deflect the debris towards the descending aorta, which carries a theoretical risk of increasing the likelihood of peripheral embolism [29].

Several different EPDs have been developed and tested for use during TAVI procedures and are summarized in Table 1.

The Sentinel Cerebral Protection System (Claret Medical/Boston Scientific, Santa Rosa, CA, USA) is a filter-based device that utilizes two filters. These filters are strategically placed in the brachiocephalic artery and the left common carotid artery, the two major vessels supplying blood to the brain [44]. The Sentinel system is delivered via a 6 Fr sheath, typically through a right radial artery access. It holds the distinction of being the most extensively studied cerebral EPD and is currently the only device of its kind that has received approval from the Food and Drug Administration (FDA) in the United States [24]. Studies have demonstrated that the Sentinel device is highly effective in capturing embolic debris, with successful capture reported in over 99% of cases. However, it is important to note that the Sentinel system does not provide protection to the left vertebral artery, which can contribute up to 20% of the total blood supply to the brain [19,45].

The TriGuard 3 device (Keystone Heart, Ltd., Caesarea, Israel/Venus Medtech Inc., Hangzhou, China) represents a deflector-based approach to embolic protection [46]. This system is placed in the aortic arch through femoral access, utilizing either an 8 Fr or a 9 Fr sheath. A key feature of the TriGuard device is its design to provide full cerebral protection by covering all three major branches of the aortic arch: the brachiocephalic artery, the left common carotid artery, and the left subclavian artery. The device employs a semi-permeable mesh that is designed to deflect particles larger than 140 µm away from the cerebral circulation and towards the descending aorta [24].

The ProtEmbo Cerebral Protection System (Protembis GmbH, Aachen, Germany) is a more recently developed cerebral EPD that also aims to protect all three cerebral vessels [47]. This device utilizes a novel design with a 60 µm pore size filter. The ProtEmbo is a temporary, single-use, intra-aortic embolic deflection filter that is used as an adjunctive device during TAVI. Notably, it is currently the only available device that can be positioned through a 6 Fr left radial access sheath [47,48].

Other EPDs that have been investigated include the Embol-X system (Edwards Lifesciences, Irvine, CA, USA), which is a filter system initially designed for use during conventional cardiac surgery and requires direct access to the ascending aorta [49]. A modified version has been tested for use in transaortic TAVI procedures, providing full cerebral coverage [24]. The Embrella deflector device (Edwards Lifesciences, CA, USA) was one of the earliest dedicated EPDs for TAVI [50]. Although no longer under development, it was used via the right radial or brachial artery and covered the brachiocephalic trunk and left common carotid artery, typically leaving the left vertebral artery unprotected [24]. The Emblok device (Innovative Cardiovascular Solutions, Grand Rapids, MI, USA) is designed to be positioned in the ascending aorta and aortic arch via a femoral artery using an 11 Fr sheath, aiming to provide circumferential protection against embolic materials originating from the aorta. Finally, the Emboliner system (Emboline, Santa Cruz, CA, USA) is advanced through a 9 Fr transfemoral sheath and is engineered to protect all three cerebral vessels as well as the entire body from embolization [51]. The Claret Montage Dual Filter System (Claret Medical Inc., Santa Rosa, CA, USA) is another filter-based device with a similar design to the Sentinel system, also positioned in the brachiocephalic and left common carotid arteries [27,52]. The variety in EPD designs, including the choice between filters and deflectors, different access routes for deployment, and the extent of cerebral vascular coverage, highlights the ongoing efforts to refine and optimize embolic protection strategies during TAVI procedures [24].

## 4. The Current Evidence for Using Specific Embolic Protection Devices During TAVI

### 4.1. Major Trials

The clinical evidence regarding the effectiveness of EPDs in TAVI has been investigated in several randomized controlled trials (RCTs). The PROTECTED TAVR trial stands out as a large, multicenter RCT that included over 3000 patients and evaluated the use of the Sentinel device [30]. The primary outcome of this trial was the rate of strokes within 72 h after TAVI or before hospital discharge. The results of the PROTECTED TAVR trial did not demonstrate a statistically significant reduction in the overall rate of clinical stroke in patients who received the Sentinel device compared to those who did not. However, there was a trend observed towards a reduction in the incidence of disabling stroke in the group that underwent TAVI with cerebral EPDs [30]. Notably, the study did not find a significant difference in mortality between the EPD group and the control group. The lack of a clear statistically significant benefit in preventing all strokes in this large trial raises important considerations regarding the routine use of the Sentinel device in all patients undergoing TAVI [30].

The British Heart Foundation’s PROTECT-TAVI trial (ClinicalTrials.gov, ID: NCT02895737) was a large-scale, randomized, multicenter study in the UK evaluating over 7700 patients undergoing TAVI with or without the Sentinel cerebral EPD. Patients were randomized to receive the device or no protection, regardless of access route. The primary endpoint—occurrence of stroke within 72 h post-procedure or before discharge—showed no statistically significant difference between groups (2.3% with CEP vs. 2.9% without; *p* = 0.30). However, disabling strokes were less frequent in the EPD group (0.5% vs. 1.3%). The device was successfully deployed in 94.4% of attempted cases, with minimal access-related complications. While the overall stroke rate did not differ significantly, the reduction in disabling stroke suggests a potential clinical benefit, reinforcing the need for individualized decision-making in cerebral EPD use during TAVI procedures [30]. The results of this trial were announced in the American College of Cardiology (ACC) 2025 Congress, in March 2025.

In contrast, the CLEAN-TAVI Trial, a single-center RCT conducted in Germany, randomized 100 patients to undergo TAVI either with or without the Claret Sentinel device [17]. This trial reported a significantly lower number of new cerebral lesions detected on post-procedure imaging in the brain areas protected by the Sentinel device compared to the control group. However, the CLEAN-TAVI trial did not report any significant differences in clinical cerebrovascular events between the two groups [17]. These findings suggest that while the Sentinel device may be effective in reducing subclinical embolic events, the direct link to a reduction in clinically evident stroke remains to be definitively established.

The DEFLECT III Trial was a multicenter RCT that evaluated the TriGuard system in a cohort of 85 patients [45]. The trial reported a lower incidence of major adverse cardiac and cerebrovascular events (MACCE) and stroke at 30 days in the group that received the TriGuard device, although these differences did not reach statistical significance. In a subgroup of patients where complete cerebral coverage was achieved with the TriGuard system, the device was associated with a higher rate of freedom from new brain lesions at 1-month follow-up and a lower degree of neurological deficit as assessed by the National Institutes of Health Stroke Scale (NIHSS) [24,45]. While these results provided early indications of potential benefits associated with the TriGuard device, particularly in terms of subclinical outcomes and neurological deficits, the small sample size of the study limits the ability to draw firm conclusions [45].

The newer generation of the TriGuard device, TriGUARD 3, was investigated in the REFLECT II Trial [53]. This RCT randomized TAVI patients to either a device arm or a control arm. The results of REFLECT II showed that the composite primary safety endpoint at 30 days and the median total new lesion volume, as assessed by DW-MRI, were similar in both the device and the control groups. These findings did not demonstrate a clear benefit of the TriGUARD 3 in reducing either the primary safety endpoint or the volume of new brain lesions, suggesting that the earlier promising results observed with the first-generation device may not be consistently replicated with the newer iteration [53].

The PROTEMBO C Trial was a single-arm study that evaluated the safety and performance of the ProtEmbo Cerebral Protection System in 41 patients [48]. The trial met its primary safety and performance endpoints when compared to pre-specified historical performance goals. Additionally, the study found that the new DW-MRI lesion volumes in patients who received the ProtEmbo device were smaller than those observed in historical data. These initial results from the PROTEMBO C Trial are promising for this novel EPD and warrant further evaluation in larger RCTs to confirm its efficacy in reducing clinical and subclinical cerebrovascular events [54].

The primary and secondary endpoints in these RCTs have varied but commonly include the incidence of stroke (both all stroke and disabling stroke), all-cause mortality, and major adverse cardiovascular events (MACE). Subgroup analyses in some studies have suggested the potential benefits of EPDs in specific patient populations. For instance, expert opinion suggests that individuals with a higher baseline risk of stroke, such as those with a history of prior stroke, renal impairment, bicuspid aortic valves, severe aortic valve calcification, those undergoing valve-in-valve procedures, patients with a porcelain aorta, and younger individuals, might derive the greatest benefit from the use of EPDs [55]. Additionally, a subgroup analysis from one meta-analysis indicated a trend towards a significant reduction in the number of new brain lesions per patient with the use of EPDs, particularly in those who received self-expanding valves [56]. These observations highlight the importance of identifying specific high-risk patient subgroups who may experience a more pronounced benefit from EPDs, allowing for a more tailored and effective approach to their utilization [11,26,55].

### 4.2. Meta-Analyses

Several meta-analyses and systematic reviews have synthesized the available evidence on the use of EPDs in TAVI, providing a broader perspective on their efficacy and safety, and are summarized in Table 2.

A meta-analysis by Harmouch et al. [12], focusing specifically on the Sentinel CPS, found no statistically significant difference in the overall risk of periprocedural stroke between patients undergoing TAVI with the Sentinel device and those without CEP. However, this analysis did find a statistically significant decrease in the risk of periprocedural disabling strokes with the use of the Sentinel CPS. Another meta-analysis by Tan et al. evaluated the efficacy and safety of the Sentinel Cerebral Protection System [57]. This study reported significant reductions in mortality, all stroke, and disabling stroke in the overall analysis of the studies included. However, when the analysis was restricted to only RCTs, a significant reduction was observed only for the incidence of disabling stroke in the Sentinel group. Furthermore, a Bayesian meta-analysis of randomized EPD trials conducted by Heuts et al. focused on the clinically relevant reduction in disabling stroke [58]. While their analysis indicated a high probability of a beneficial treatment effect with EPDs, they concluded that this effect was unlikely to be clinically relevant based on predefined criteria.

Reddy et al. performed a meta-analysis of RCTs comparing EPDs with control during TAVI [59]. Their findings showed no significant differences between the use of EPDs and the control group for the outcomes of all stroke, disabling stroke, non-disabling stroke, or all-cause mortality. However, in a subgroup analysis restricted to the Sentinel EPD, they observed a significant reduction in the risk of disabling stroke. In contrast, a meta-analysis by Elfaituri et al. of RCTs found that the use of CEP devices during TAVI did not significantly alter the total lesion volume or reduce the risk of stroke, disabling stroke, or overall mortality [60]. Notably, Ndunda et al. conducted a meta-analysis that reported significant reductions in mortality, all stroke, and disabling stroke with the use of the Sentinel device during TAVI [61].

Similarly, Testa et al. found that the use of EPDs in TAVI was associated with a reduction in MACCE, mortality, and stroke [62]. A meta-analysis by Pagnesi et al. indicated that the use of EPDs during TAVI may be associated with a smaller volume of silent ischemic lesions and a smaller total volume of such lesions [16]. However, this analysis also suggested that EPDs may not reduce the number of new lesions, and the quality of evidence for a reduction in clinically evident stroke or 30-day mortality was very low. Finally, Wang et al. performed a meta-analysis that examined the efficacy of different types of cerebral EPDs [63]. Their study found that I&LCCA (devices covering the innominate and left common carotid arteries)-type EPDs could reduce the risk of stroke within 30 days following TAVI, but TMCA (devices covering the three major cerebral arteries)-type EPDs did not show a similar benefit.

Overall, the findings from these multiple meta-analyses present a mixed picture regarding the clinical benefit of EPDs in TAVI. While some meta-analyses suggest a potential reduction in disabling stroke, particularly with the Sentinel device, the evidence for a consistent reduction in all-cause stroke or mortality is less robust. Furthermore, the presence of heterogeneity across studies, often due to variations in study design, patient populations, and the specific EPDs evaluated, along with the limitations of many RCTs having small sample sizes and being underpowered for clinical outcomes, contributes to the challenges in drawing definitive conclusions [24]. The varying results underscore the need for larger, well-designed RCTs with consistent endpoints to more definitively determine the clinical efficacy of EPDs in TAVI.

## 5. Current Controversies

### 5.1. Complete vs. Partial Cerebral Embolic Protection

Current evidence theoretically favors EPDs offering complete cerebral coverage during transcatheter procedures, particularly given that the left vertebral artery contributes up to 20% of total cerebral blood flow. The volume and size of embolic debris passing through the left vertebral artery during TAVI procedures appear comparable to that seen in the brachiocephalic trunk and left common carotid artery. Consequently, clinical and imaging outcomes (such as MRI-detected lesions and cognitive function) may be superior with comprehensive cerebral protection versus partial protection, but prospective RCTs are required to validate this hypothesis definitively [55].

### 5.2. Deflection vs. Capture of Debris During Transcatheter Cardiac Interventions

Similarly, the concept of capturing and extracting debris generated during transcatheter interventions seems intuitively advantageous compared to deflecting particles away from cerebral circulation and towards peripheral arteries. Nonetheless, the current literature has not demonstrated superiority in terms of clinical endpoints, including lower incidence of distal organ damage or limb ischemia, with debris capture versus deflection techniques. Moreover, it remains crucial to consider the trade-off between potential benefits of debris capture and risks related to the larger vascular access necessary for such devices, including higher rates of vascular complications [43]. An emerging area where these comprehensive debris-capturing systems could become increasingly relevant involves procedures designed to remove or resect native or prosthetic valve leaflets, known to carry a high embolic risk. Additionally, given comparable stroke risks during transcatheter mitral valve edge-to-edge repair procedures (which typically utilize large-bore venous rather than arterial access), avoiding large arterial access devices would be particularly beneficial if cerebral EPDs eventually demonstrate clear utility in these contexts [64,65].

### 5.3. EPD Use During Non-Femoral TAVI Procedures

The expanding application of TAVI in patients with challenging iliofemoral anatomies—characterized by severe calcification or tortuosity incompatible with current delivery systems—has led to an increased adoption of alternative (non-femoral) access approaches. Currently, no specific guidelines exist regarding optimal EPD selection based on vascular access routes [11]. The choice of an embolic protection device in these scenarios should carefully consider the technical compatibility with various TAVI access methods. Specifically, particular caution is warranted when radial-access EPDs are utilized in patients undergoing transcarotid, -axillary, or -subclavian TAVI, or when femoral-access EPDs are employed in patients undergoing transcaval or transaortic approaches [66]. Device efficacy and safety may be impacted significantly by interaction with TAVI delivery catheters and procedural adaptations specific to non-femoral vascular approaches [11,55].

### 5.4. EPD Use During Surgical Aortic Valve Replacement

Stroke rates following surgical aortic valve replacement (SAVR) procedures vary widely, ranging from approximately 1% to as high as 17% [67]. A propensity-matched analysis comparing SAVR and TAVI patients from the PARTNER trials demonstrated a higher incidence of major strokes at 30 days after SAVR compared to TAVI (3.9% vs. 2.2%, respectively), accompanied by significant quality-of-life deterioration at one year in patients who experienced a stroke [68]. Moreover, silent brain infarctions occur frequently during SAVR, reportedly affecting up to 60% of patients. Despite this, an RCT assessing two cerebral protection approaches (a suction-based debris extraction system and an intra-aortic filter device) versus a control group using standard aortic cannulation during SAVR did not demonstrate a significant reduction in clinical stroke or imaging-identified cerebral infarctions, even though these protection devices effectively captured embolic material [69]. Thus, although potentially appealing, the application of transcatheter EPDs in the surgical valve replacement context has yet to be explored or reported in the literature.

## 6. Emerging Cerebral Embolic Protection Technologies in TAVI

The evolution of cerebral EPDs continues, with several next-generation systems under clinical development aiming to enhance cerebral coverage, improve safety, and optimize procedural integration. Below are selected devices currently in preclinical or early clinical phases.

### 6.1. Emblok Embolic Protection System

The Emblok system (Innovative Cardiovascular Solutions) is engineered to provide complete circumferential shielding of the aortic arch, thereby protecting all supra-aortic vessels. It is deployed via an 11 Fr single-access sheath compatible with a 0.035” guidewire. An integrated 4 Fr radiopaque pigtail catheter facilitates continuous fluoroscopic visualization and aortography for both valve and cerebral EPD deployment. The conical filter, made from polyurethane mesh (pore size 125 μm) and supported by a nitinol frame, is positioned proximally to the brachiocephalic artery. The system is deployed after positioning the valve in the ascending aorta and is retracted after valve implantation to allow for device retrieval. Early human use has demonstrated procedural feasibility [70].

### 6.2. ProtEmbo Cerebral Protection System

The ProtEmbo device (Protembis GmbH) is a low-profile, intra-aortic deflection filter designed to be inserted via a 6 Fr left radial or brachial access. It uses a self-expanding nitinol frame covered by a heparin-coated mesh with ultra-fine 60 μm pores—currently the smallest among EPDs in development. Radiopaque markers assist in optimal positioning to protect all three supra-aortic vessels. The device is fully retrievable, and deployment is performed using a straightforward handle-based interface. Initial feasibility was demonstrated in the PROTEMBO SF trial (n = 4), and interim results from the PROTEMBO C trial (n = 41) have shown encouraging safety and performance outcomes. A large-scale pivotal trial (ProtEmbo IDE) involving up to 500 patients in Europe and the U.S. began enrollment in the third quarter of 2023 [54,71].

### 6.3. Emboliner Total Embolic Protection Device

The Emboliner device (Emboline Inc.) is a cylindrical mesh filter designed to encircle the aortic arch and shield all three major cerebral arteries. Constructed with a nitinol frame and 125 μm pore mesh, the device aims to capture and remove embolic debris generated during TAVI. Its 10 Fr delivery system includes a 6 Fr integrated pigtail catheter and a port to accommodate TAVI delivery systems. The ongoing SAFE-PASS program has tested its performance [51], and a pivotal RCT comparing Emboliner to Sentinel (Clinical Trials ID: NCT05684146) is currently underway. This U.S.-based study will enroll 500 TAVI patients and aims to evaluate the 30-day composite major adverse cardiac and cerebrovascular events (MACCE) rate—defined as all death, stroke and Stage 3 acute kidney injury—evaluated on a per-patient basis, post-TAVI.

### 6.4. CAPTIS Embolic Protection System

The CAPTIS system offers a hybrid mechanism by deflecting embolic material in the aortic arch while simultaneously capturing debris in the descending aorta. It consists of a PEEK mesh filter (115 × 145 μm pores) supported by a nitinol frame configured to conform to the arch anatomy and descending aortic wall. Anchoring elements and circumferential collector pockets enhance device stability and debris retention. The 16 Fr system is delivered via the same femoral access as TAVI, avoiding additional puncture sites. First-in-human data were presented at the EuroPCR 2022 conference [72].

### 6.5. FLOWer Embolic Protection Device

Previously known as the Embrace filter, the FLOWer device is designed to protect both cerebral and peripheral vasculature. It comprises a cylindrical mesh with 70 μm pores and a frame tailored to adapt to different aortic arch morphologies. Available in three sizes, the system is compatible with all major TAVI platforms. The 12 Fr delivery sheath incorporates a 5 Fr pigtail catheter and a dedicated port. In vitro testing suggested a 99% capture rate for emboli >150 μm in the arch and 84% in downstream systemic circulation. Preliminary results from the NAUTILUS first-in-human study (Clinical Trials ID: NCT04704258) were presented at EuroPCR 2023 [28].

### 6.6. POINT-GUARD Dynamic Cerebral Protection System

The POINT-GUARD device is a second-generation embolic protection system aiming for comprehensive aortic arch and cerebral vessel coverage. It features a highly adaptable filter mesh supported by a dynamic frame with proximal and distal anchoring mechanisms, dual-edge perimeter sealing, and a proximal debris capture pouch. Its asymmetric design enables it to accommodate complex aortic arch geometries and ensure full anterior–posterior coverage. The first-generation model was evaluated in the POINT-GUARD First-In-Human CENTER study (n = 4), but data are not yet publicly available and were presented at TCT 2019 in San Francisco [73].

## 7. Future Directions and Contemporary Clinical Practice

At present, there is insufficient high-quality evidence to justify the universal application of cerebral EPDs in all patients undergoing TAVI. Moreover, there remains a lack of definitive guidance on which patient subsets may derive the most benefit [74,75]. The ability to draw strong conclusions about the clinical effectiveness of EPDs is further hindered by the low incidence of stroke in many studies, reducing statistical power. Nevertheless, several ongoing and planned clinical trials may provide the clarity needed to inform selective and evidence-based EPD use in TAVI.

Following the recent results of the PROTECT-TAVI trial, a pooled patient-level meta-analysis combining data from the PROTECTED TAVR and BHF PROTECT-TAVI trials (registered under PROSPERO: CRD42022324160) is expected to include more than 10,000 patients. This collaboration aims to generate more definitive evidence on the efficacy of the Sentinel device in stroke prevention. However, these findings will specifically pertain to the Sentinel system and may not be generalizable to other EPD technologies, underlining the need for device-specific validation.

Several other clinical studies are currently underway, seeking to define the role of newer-generation cerebral EPDs. The ProtectH2H (Clinical Trials ID: NCT05684146) trial, a pivotal randomized comparison between the Emboliner device and the Sentinel system, is set to assess 30-day major adverse cardiovascular and cerebrovascular events (MACCE) in a cohort of 500 patients. Another promising study is the PROTEMBO trial (Clinical Trials ID: NCT05873816), which is evaluating the ProtEmbo system against a hybrid control group (comprising both no protection and SENTINEL). The trial includes patients with severe symptomatic native aortic stenosis undergoing TAVI and is projected to complete in 2025.

Likewise, the EMBLOK study (Clinical Trials ID: NCT05295628) is conducting a head-to-head comparison between the EMBLOK device and SENTINEL, aiming to assess safety, performance, and efficacy during TAVI. Completion is anticipated in 2025. The NAUTILUS trial (Clinical Trials ID: NCT04704258) is focused on the FLOWer system, investigating its ability to reduce cerebral embolization during TAVI procedures. It will evaluate both clinical outcomes and device performance metrics.

These next-generation devices—designed to provide full cerebral and even systemic protection—differ in key technical aspects such as vascular access route, delivery sheath size, and filter mesh configuration. The ideal embolic protection system should ensure complete cerebral coverage, be easy to deliver and position, maintain procedural stability, and above all, demonstrate a favorable balance of safety and clinical efficacy [13].

Despite incremental technological improvements, the clinical community remains divided regarding the routine use of EPDs. While some interventionalists advocate for their use, citing minimal added procedure time and potential stroke reduction, others remain skeptical due to inconsistent trial results and additional procedural complexity and costs. This uncertainty has led many to adopt a selective approach, focusing EPD use on patients perceived to be at elevated risk for cerebral embolization.

Identifying these high-risk patients, however, is still challenging. Even large trials such as PROTECTED TAVR and PROTECT-TAVI failed to consistently demonstrate benefit in predefined subgroups. Other barriers include the cost of the devices, the need for additional access sites (which may increase vascular complication rates), and the technical demands of navigating and positioning the device in the aortic arch [11]. Consequently, the adoption of EPDs remains limited. According to the STS/ACC TVT Registry, the Sentinel system was used in just 7.1% of TAVI procedures across over 550 U.S. centers between 2018 and 2019. Reimbursement policies vary widely, but data suggest that institutional case volume, rather than reimbursement rates, is a more significant driver of cerebral EPD adoption [76]. Nonetheless, caution is warranted when interpreting registry data, as these figures cannot necessarily be generalized to all practice settings.

In practice, cerebral embolic protection is most likely to translate into a clinically meaningful benefit when both the embolic load is expected to be high and the consequences of even a small infarct would be substantial. Accordingly, operators should give strongest consideration to EPD use in patients with a previous stroke or transient ischemic attack (particularly within the preceding year); those with severe annular, leaflet, or ascending-aorta calcification (“porcelain arch”) that is prone to fragment during valve manipulation; anatomically challenging arches or horizontal aortas that necessitate prolonged catheter steering; valve-in-valve or redo TAVI cases where fractured bioprosthetic leaflets add embolic debris; bicuspid valves in relatively young recipients where the eccentric calcium burden raises risk and the lifetime impact of disability is greatest; and individuals with a high intrinsic thrombo-embolic profile—namely, atrial fibrillation or a CHA_2_DS_2_-VASc score ≥ 4. In straightforward, low-risk tricuspid anatomies lacking these features, routine EPD deployment remains discretionary until outcome-driven data mature further.

As a result, the field of embolic protection during TAVI continues to evolve, with ongoing clinical trials and potential advancements in EPD technology. Further research is warranted to explore the hypothesis of a relative benefit associated with EPDs as the technology continues to advance. Future studies should focus on identifying specific subgroups of TAVI patients who are at a particularly increased baseline risk of stroke and might therefore benefit most from EPDs [52]. Moreover, the development of new generation EPDs with improved design and efficacy is a key area of focus. Given the unique characteristics of the various cerebral EPDs currently under development, specific clinical trials tailored to their individual designs will be necessary to further advance the field of embolic protection. Beyond TAVI, the role of cerebral EPDs in other non-TAVI transcatheter heart interventions is also an emerging area of investigation. Several unanswered questions remain, including the long-term clinical consequences of silent cerebral lesions, the optimal criteria for selecting patients who would benefit most from EPDs, the comparative effectiveness of different EPDs, the impact of EPDs on cognitive function, and the overall cost-effectiveness of routine versus selective EPD use. Continued research efforts are crucial for addressing these questions and further refining the role of EPDs in TAVI, ultimately aiming to improve patient outcomes [77].

## 8. Conclusions

In conclusion, the current evidence suggests that embolic protection devices in TAVI are safe and technically feasible for deployment. These devices have demonstrated effectiveness in capturing embolic debris released during the procedure and in reducing the volume of silent cerebral ischemic lesions as detected by MRI. However, the evidence regarding the prevention of overt strokes with EPDs remains mixed. While some meta-analyses indicate a potential benefit in reducing disabling stroke, particularly with the Sentinel device, large-scale RCTs have not consistently shown a significant reduction in the overall incidence of stroke. Therefore, the routine use of EPDs in all patients undergoing TAVI is not yet clearly supported by the available evidence. A more selective approach, focusing on patients at higher risk of stroke, warrants further consideration and investigation. Continued research and ongoing technological advancements in EPD design are essential to optimize embolic protection strategies and ultimately improve the outcomes of TAVI procedures.

## Figures and Tables

**Table 1:** Overview of cerebral embolic protection devices and their key features.

Device		Cerebral Embolic Protection	Function	Pore Size	Access Site	Access Size
ProtEmbo Cerebral Protection System	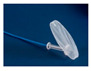	Complete	Deflect	60 μm	Left radial	6 Fr
Sentinel Cerebral Protection System	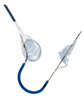	Partial	Partial capture	140 μm	Right radial	6 Fr
EMBOL-X Device	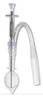	Complete	Capture and removal	120 μm	Middle sternotomy	14 Fr
TriGUARD 3 Cerebral Protection Device	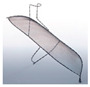	Complete	Deflect	115 × 145 μm	Femoral	8 Fr
Embrella Embolic Deflector	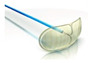	Partial	Deflect	100 μm	Radial/ brachial	6 Fr
Emblok Embolic Protection System	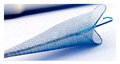	Complete	Capture and removal	125 μm	Femoral (contralateral)	11 Fr
Emboliner Total Embolic Protection	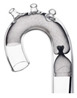	Complete	Capture and removal	150 μm	Femoral (contralateral)	10 Fr
POINT-GUARD	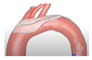	Complete	Deflect	105 µm	Femoral	10 Fr
CAPTIS Full-Body Embolic Protection Device	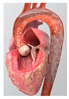	Complete	Capture and removal	115 × 145 μm	Femoral	16 Fr
FLOWer	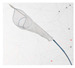	Complete	Capture and removal	70 μm	Femoral	12 Fr

**Table 2:** Key findings of major meta-analyses on embolic protection devices in transcatheter aortic valve implantation.

First Author and Year	Device(s) Evaluated	Patient Population	Primary Outcomes	Main Results	Conclusions
Harmouch et al. (2024) [12]	Sentinel CPS	TAVI patients	Periprocedural stroke	No difference in overall stroke, decreased disabling stroke (RR 0.41, *p* = 0.02)	Sentinel CPS associated with decreased disabling stroke
Tan et al. (2024) [57]	Sentinel CPS	TAVI patients	Stroke, mortality	Overall: reduced mortality, all stroke, disabling stroke. Randomized data: reduced disabling stroke	Sentinel CPS associated with lower rates of mortality, all stroke, and disabling stroke (overall), disabling stroke (randomized)
Heuts et al. (2024) [58]	Various EPD	TAVI patients	Disabling stroke	RR 0.56 (95% CI, 0.28 to 1.19), unlikely clinically relevant	Beneficial EPD effect likely not clinically relevant for disabling stroke
Reddy et al. (2023) [59]	Various EPD	TAVI patients	Stroke, mortality	No significant difference in all stroke, disabling stroke, non-disabling stroke, or mortality. Sentinel reduced disabling stroke	No significant difference in clinical endpoints with EPDs overall
Elfaituri et al. (2023) [60]	Various EPD	TAVI patients	Total lesion volume, stroke, mortality	No significant difference in total lesion volume, stroke, disabling stroke, or mortality	EPDs did not significantly alter important clinical outcomes
Ndunda et al. (2020) [61]	Sentinel CPS	TAVI patients	Mortality, all stroke, disabling stroke	Significant reductions in mortality, all stroke, and disabling stroke	Sentinel CPS associated with improved outcomes
Testa et al. (2018) [62]	Various EPD	TAVI patients	MACE, mortality, stroke	Reduced MACE, mortality, and stroke with EPD use	EPD use associated with better clinical outcomes
Pagnesi et al. (2016) [16]	Various EPD	TAVI patients	Clinically evident stroke, mortality, silent lesions	Smaller volume of silent lesions, no reduction in number of lesions, very low evidence for clinical stroke/mortality reduction	EPDs may reduce silent lesion volume but lack strong evidence for clinical stroke/mortality benefit
Wang et al. (2024) [63]	Various EPD	TAVI patients	Stroke	I&LCCA type reduced stroke risk, TMCA type did not	I&LCCA EPDs may be more effective than TMCA EPDs

Abbreviations: CPS: cerebral protection system, EPD: embolic protection devices, TAVI: transcatheter aortic valve implantation, MACE: major adverse cardiovascular events, RR: risk ratio, I&LCCA: embolic protection devices covering the innominate and left common carotid arteries, TMCA: embolic protection devices covering the three major cerebral arteries.
